# The subcellular localization of IGFBP5 affects its cell growth and migration functions in breast cancer

**DOI:** 10.1186/1471-2407-9-103

**Published:** 2009-04-03

**Authors:** Mustafa Akkiprik, Limei Hu, Aysegul Sahin, Xishan Hao, Wei Zhang

**Affiliations:** 1 Department of Pathology, The University of Texas M. D. Anderson Cancer Center, Houston, Texas, USA; 2 Marmara University, School of Medicine, Department of Medical Biology, Istanbul, Turkey; 3 Tianjin Cancer Institute and Hospital, Tianjin Medical University, Tianjin, PR China

## Abstract

**Background:**

Insulin-like growth factor binding protein 5 (IGFBP5) has been shown to be associated with breast cancer metastasis in clinical marker studies. However, a major difficulty in understanding how IGFBP5 functions in this capacity is the paradoxical observation that ectopic overexpression of IGFBP5 in breast cancer cell lines results in suppressed cellular proliferation. In cancer tissues, IGFBP5 resides mainly in the cytoplasm; however, in transfected cells, IGFBP5 is mainly located in the nucleus. We hypothesized that subcellular localization of IGFBP5 affects its functions in host cells.

**Methods:**

To test this hypothesis, we generated wild-type and mutant IGFBP5 expression constructs. The mutation occurs within the nuclear localization sequence (NLS) of the protein and is generated by site-directed mutagenesis using the wild-type IGFBP5 expression construct as a template. Next, we transfected each expression construct into MDA-MB-435 breast cancer cells to establish stable clones overexpressing either wild-type or mutant IGFBP5.

**Results:**

Functional analysis revealed that cells overexpressing wild-type IGFBP5 had significantly lower cell growth rate and motility than the vector-transfected cells, whereas cells overexpressing mutant IGFBP5 demonstrated a significantly higher ability to proliferate and migrate. To illustrate the subcellular localization of the proteins, we generated wild-type and mutant IGFBP5-pDsRed fluorescence fusion constructs. Fluorescence microscopy imaging revealed that mutation of the NLS in IGFBP5 switched the accumulation of IGFBP5 from the nucleus to the cytoplasm of the protein.

**Conclusion:**

Together, these findings imply that the mutant form of IGFBP5 increases proliferation and motility of breast cancer cells and that mutation of the NLS in IGFBP5 results in localization of IGFBP5 in the cytoplasm, suggesting that subcellular localization of IGFBP5 affects its cell growth and migration functions in the breast cancer cells.

## Background

Insulin-like growth factor (IGF) binding protein-5 (IGFBP5) is the most evolutionarily conserved member in a family of 6 high-affinity IGF-binding proteins [1,2]. IGFBP5 has been shown to have a potential role in carcinogenesis that involves 2 main pathways [3,4]. One is the IGF-dependent pathway. The other is the IGF-independent pathway, which is more complicated and less understood than the IGF-dependent pathway. Recently, Duan and colleagues showed that IGFBP5 promotes cell differentiation by regulating IGF-II actions [5]. Many of the recently published studies of IGFBP5 have focused on the involvement of the protein in apoptosis, protein-protein interaction, cell motility, cell survival, and cellular trafficking [6]. C-terminal domain of IGFBP5 contains a region known as the nuclear localization sequence (NLS; at amino acids 201 to 218)) [7,8] that is responsible for the nuclear transport of IGFBP5. The NLS contains a heparin-binding motif (HBM, consensus sequence BBBXXB, where B is a basic amino acid and X is any amino acid) at amino acids 206 to 211 (KRKQCK) and carries alternative IGF-binding sites [9,10]. Therefore, this region seems to be critical in determining the diverse functions of IGFBP5. It has been shown that IGFBP5 stimulates cell migration through interaction with cell surface heparan sulfate proteoglycans [11] and this activity is negatively regulated by fibronectin [12]. IGFBP5 has been shown to determine cell fates by regulating apoptotic molecules (bax, bcl-2) [13] and activating p38 MAP kinase and Erk 1/2 signal transduction pathways [14]. Reports also show that IGFBP5 regulates gene transcriptions [15]. Thus, IGFBP5 has diverse functions in different cellular compartment.

Using cDNA and tissue microarray technologies, we and others have found that IGFBP5 overexpression is associated with a poor prognosis and with metastasis in patients with breast cancer [16-19]; however, the mechanism by which IGFBP5 promotes metastasis is unknown. Results from gain-of-function studies conducted via forced expression of IGFBP5 in breast cancer cell lines has shown a surprising inhibitory effect of IGFBP5 on cell proliferation [13,20]. In these *in vitro *studies, IGFBP5 was found to be localized to the nucleus [7,8]. In contrast, immunohistochemistry studies of breast cancer tissues have shown IGFBP5 to be localized mainly to the cytoplasm [18,19]. These findings suggest that the cellular localization of IGFBP5 determines whether it has a stimulatory or inhibitory effect on cells.

To test our hypothesis that subcellular localization of IGFBP5 affects its functions in host cells, we generated a construct containing a deleted form of IGFBP5 in which the last 5 amino acids in the NLS were deleted. Next, we established cell lines overexpressing either the wild-type or the mutant form of IGFBP5 using MDA-MB-435 as the parental cell line and investigated the effects of the wild-type and mutant proteins on cell proliferation and motility. To visualize the subcellular localization of IGFBP5, we generated IGFBP5-pDsRed fluorescence fusion constructs containing either wild-type or mutant IGFBP5. Deletion of the last 5 amino acids of NLS in IGFBP5 eliminated nuclear localization of IGFBP5 and significantly promotes breast cancer cell proliferation and motility.

## Methods

### Plasmid construction

**Figure 1:**
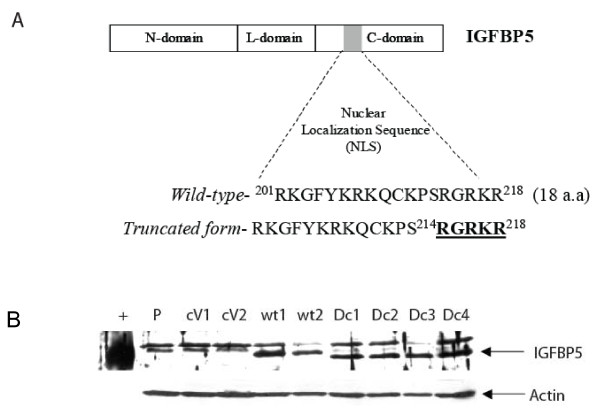
**Establishment of the wild-type and mutant forms of IGFBP5 stably expressing cell lines**. A. Schematic diagram of the IGFBP5 cDNA fragments used to generate the IGFBP5 expression constructs. Wild-type IGFBP5 contains a NLS located at the C-terminal domain of the protein from amino acid 201 to 218 and indicated by a gray solid square. For the mutant form of IGFBP5, 5 amino acids in the NLS from 214–218 (black characters) were deleted. B. Western blot analysis of IGFBP5 expression. MBA-MD-435 breast cancer cells were stably transfected with either the wild-type or the mutant forms of IGFBP5 construct and the whole cell lysate from parental MBA-435 cells (P) and different stable lines were subjected to western blot analysis (40 μg/lane) using an anti-human IGFBP5 antibody. The blot was re-probed with actin to normalize for protein loading. Neither parental nor vector-transfectant (V) cells had detectable levels of IGFBP5. 10 ng of purchased human recombinant IGFBP5 protein was served as a positive control (+). wt, wild-type and d, deletion form IGFBP5.

The full-length IGFBP5 cDNA was generated by reverse transcriptase-polymerase chain reaction (RT-PCR) using total RNA from MCF7 breast cancer cell line. The primer sequence was: forward primer, 5'-GCCACCATGGTGTTGCTCACCGCGGTCCTCCTGC-3'; and reverse primer, 5'-TCACTCAA-CGTTGCTGCTGTCGAAGGTGTG-3'. A Kozak consensus GCCACC is inserted in front of the IGFBP5 translation initiation site to increase the translation efficiency. The PCR product was inserted into pCR^®^2.1TOPO plasmid (Invitrogen, Carlsbad, CA), and several clones were chosen for DNA sequencing. The IGFBP5 cDNA with the wild-type sequence was digested from the TA-cloning plasmid using EcoRI restriction enzyme and was inserted into the EcoRI site of the pcDNA3.1(+) expression vector (Invitrogen) to obtain the IGFBP5wt/pcDNA3.1(+) construct. Again, several clones were sequenced, and the clone with right orientation and the cDNA sequence was chosen to perform cell transfection to generate wild-type IGFBP5-overexpressing cell lines. The mutant IGFBP5 expression construct [IGFBP5mt/pcDNA3(+)] was generated using the QuickChange Kit (Stratagene, La Jolla, CA) using the IGFBP5wt/pcDNA3.1(+) expression plasmid as a template. As shown in Figure 1A, 5 amino acids in the NLS (214 to 218; black characters) were deleted. Several clones were sequenced, and the clone with right orientation and IGFBP5 cDNA with the mutant sequence was chosen to perform the transfection and to generate mutant IGFBP5-overexpressing cell lines.

To generate wild-type and mutant IGFBP5 fluorescence fusion proteins, we used the same upstream primer used to generate the wild-type IGFBP5 expression construct. To make the fusion protein in the reading frame, a new downstream primer was generated using the following sequence: 5'-CCGGAATTCCGGACTCAACGTTGCTGCTGTCGAAGG-TGTG-3'. The PCR products were generated using the IGFBP5wt/pcDNA3.1(+) and the IGFBP5mt/pcDNA3.1(+) plasmids to generate wild-type and mutant IGFBP5-fluorecence fusion proteins (respectively). The PCR products were inserted into the pDsRed2-N1 vector (BD Biosciences, San Jose, CA) at the EcoRI restriction enzyme site after digestion with the same enzyme to obtain the IGFBP5wt/pDsRed2-N1 and the IGFBP5mt/pDsRed2-N1 constructs.

### Generation of stably transfected cell lines

MDA-MB-435 cells were grown at 70% confluence and transfected with the pcDNA3.1(+) vector alone, with IGFBP5wt/pcDNA3.1(+), and with IGFBP5mt/pcDNA3.1(+) using Nucleofector (Amaxa, Gaithersburg, MD). The transfection was performed with Solution T and program T_20_. Selection of stable cell clones was carried out in the presence of 600 μg/ml G418 (Invitrogen) in culture medium at 48 h post-transfection, and the cells were re-fed every 3 days. Several single clones were selected and expanded for 2–3 weeks, and the IGFBP5 expression level was detected by western blot analysis.

### Analysis of BrdU incorporation

The cell growth rates of the vector and of the wild-type and the mutant IGFBP5 transfectants were measured by bromodeoxyuridine (BrdU) incorporation using BrdU flow kits (BD Biosciences) per the manufacturer's instructions. Briefly, the cells were cultured in complete medium for 24 h and then incubated in fresh complete medium with 10 μM BrdU for 2 h. The incorporated BrdU was visualized by immunofluorescence staining using an anti-BrdU antibody conjugated with fluorescein isothiocyanate. The nuclei were counterstained with DAPI, and images were taken at a magnification of 63×. Cells not treated with anti-BrdU antibody served as negative controls. The number of total cells and the BrdU-positive cells were counted from 3 fields of each group under a fluorescence microscope, and the quantification data were graphed as a ratio of the number of BrdU-positive cells to the number of total cells. The geometric mean fluorescence intensity from the same cell population was recorded by fluorescence-activity cell sorter (FACS) analysis. The fluorescence intensity from the negative control was graphed as zero, and the fluorescence intensity from the rest of the samples became the value remaining after the fluorescence intensity was subtracted from the negative control.

### Cell culture and fluorescence microscopy imaging

The MDA-MB-435 breast cancer cell line was obtained from the American Type Culture Company (ATCC; Menassas, VA) and was routinely maintained in Dulbecco's modified Eagle's medium/F-12 supplemented with 10% fetal bovine serum (FBS) and 1% antibiotic cocktail under standard conditions. For microscopy imaging, 2 × 10^4 ^IGFBP5wt/pDsRed2-N1 or IGFBP5mt/pDsRed2-N1 fusion construct-transfected cells were seeded per cell into glass slide chambers (BD Pharmingen) and allowed to attach for 40 h. The cells were then fixed with 4% paraformaldehyde in phosphate-buffered saline. After washing, the cells were counterstained with DAPI. Images were taken using an Axioplan 2 fluorescence microscope (ZEISS, Oberkochen, Germany) at 63× magnification.

### In vitro migration assay

Falcon cell-culture inserts containing an 8-μ pore PET membrane (BD Biosciences) were used to perform the *in vitro *migration assay. The vector-alone transfectants and the IGFBP5wt/pcDNA3.1(+) and IGFBP5mt/pcDNA3.1(+) stable cells were seeded into the upper chambers at a density of 2 × 10^4 ^cells/well in 500 μl of serum-free medium. To the lower chambers, 1 ml of 10% FBS-containing medium was added. The plates were then incubated for 20 h at 37°C with 5% CO_2_. The unmigrated cells in the upper chambers were gently removed using a cotton swab. Cells that had migrated through the membrane pore into the lower sides of the filters were fixed, stained with HEMA-DIFF solution (Fisher Scientific, Philadelphia, PA), and counted under a regular light microscope.

### Western blot analysis

IGFBP5 in the whole-cell lysate and conditioned media (CM) was detected according to a previously describe standard protocol [21]. Briefly, cell lysates from different cell populations were collected, and protein concentration was measured using the Bio-Rad protein assay (Bio-Rad Laboratories, Hercules, CA). Equal amounts of proteins were then loaded into 10% sodium dodecyl sulfate-polyacrylimide gels and resolved by electrophoresis. After transfer to nitrocellulose membranes, it was probed with anti-IGFBP5 antibody (diluted 1:1000 in TBST, 5% non-fat dry milk; Santa Cruz Biotechnology, Santa Cruz, CA). Bands were detected using the chemiluminescence detection method (Amersham Biosciences, Piscataway, NJ) and exposed on x-ray film. The IGFBP5 level in the same volume of conditioned media from the same cell populations of similar densities for the cell lysate was measured simultaneously.

### Statistical analysis

Data are expressed as the mean ± standard error. Statistical analysis was performed using the Student's *t *test. Differences in means were evaluated by a 2-tailed *t *test assuming unequal variances. A p value of ≤ 0.05 was considered statistically significant.

## Results

### Deletion of amino acids 214 to 218 of IGFBP5 altered subcellular localization of the protein in the MDA-MB-435 breast cancer cells

**Figure 2:**
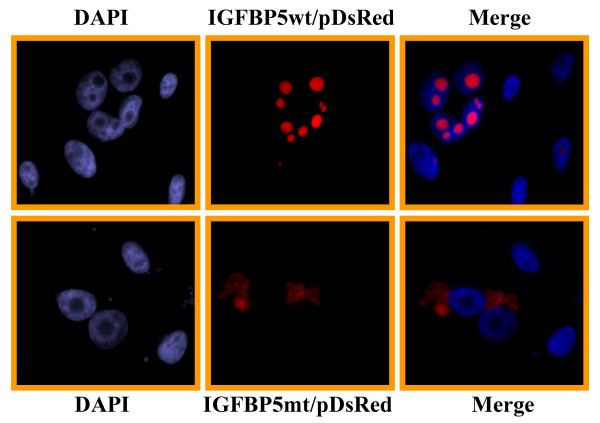
**Deletion of amino acids from 214 to 218 of IGFBP5 protein eliminates its nuclear localization**. The wild-type (IGFBP5wt/pDsRed2-N1) or mutant (IGFBP5mt/pDsRed2-N1) fusion construct was transiently transfected into MBA-MD-435 breast cancer cells. The cells were fixed 48 h post-transfection and counterstained with DAPI. The images were taken by a fluorescence microscope at 63× magnification.

**Figure 3:**
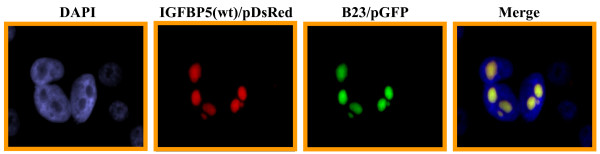
**Wild-type IGFBP5 mainly localized at the cellular nucleolus**. IGFBP5 wt/pDsRed2-N1 and B23/pGFP constructs were co-transfected into MBA-MD-435 cells. After 48 h post-transfection, the cells were fixed and counterstained with DAPI. The IGFBP5 fusion protein showed co-localization with B23, which is a marker for the nucleolus.

**Figure 4:**
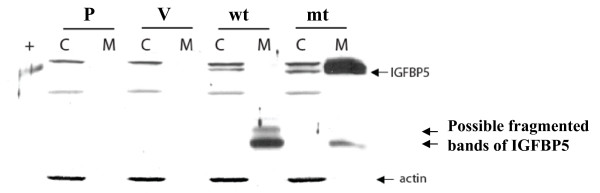
**Western blot analysis of IGFBP5 expression in both cell lysate and conditioned medium**. One million cells were cultured in 100-mm diameter tissue culture dish in the complete medium for 24 h followed by an additional 24-h incubation in the serum-free medium. Both cell lysate and conditioned medium were collected. The conditioned medium was concentrated 3 times using YM-10 column (Millipore) and 40 μg of protein from the cell lysate and 45 μl of the concentrated conditioned medium were subject for western blot analysis. The blot was re-probed with anti-actin to normalize for protein loading. wt, wild-type and mt, mutant form IGFBP5.

To determine whether the nuclear localization sequence is required and sufficient for transporting IGFBP5 into the nucleus, we constructed a mutant IGFBP5 expression vector in which 5 amino acids in the NLS were deleted (Figure 1A). We transfected both wild-type and mutant IGFBP5 expression vectors into the MDA-MB-435 breast cancer cells, and established several stable cell lines overexpressing these vectors. IGFBP5 expression was detected in the cells by western blot analysis using whole-cell lysates (Figure 1B). The parental cells and the vector-alone transfectants were used as controls. In order to visualize the subcellular localization of wild-type and mutant IGFBP5 in the cells, we generated constructs that fused fluorescent pDesRed on the C-terminal region of either wild-type IGFBP5 or mutant IGFBP5. After transfection of the expressing vectors, we observed that wild-type IGFBP5 was mostly localized in the nucleolus. The nucleolus localization of wild-type IGFBP5 was confirmed by the observation of colocalization of IGFBP5 and the known nucleolus protein B23 [22] (Figure 2). Mutated IGFBP5 was not detected in the nucleolus but was detected at a low level in the cytoplasm (Figure 3). To determine whether the low cytoplasmic level of mutant IGFBP5 was the result of increased secretion of the IGFBP5 into the media, we collected both cell lysate and conditional media from the stable cell lines and performed western blot analysis. Again, we could not detect IGFBP5 from either the cell lysates or the conditioned media in the parental and vector-transfected cells (Figure 4). We did observe overexpression of IGFBP5 from the IGFBP5wt and IGFBP5mt stable clones, and the cells overexpressing the mutant form IGFBP5 showed a much higher level of IGFBP5 in the conditioned medium compared with the wild-type IGFBP5 transfectants (Figure 4). Interestingly, we observed that the majority of the secreted wild-type IGFBP5 was cleaved whereas that majority of the mutant IGFBP5 remained intact in the conditioned media (Figure 4).

### Deletion of amino acids 214 to 218 of IGFBP5 protein promoted proliferation of MDA-MB-435 breast cancer cells

**Figure 5:**
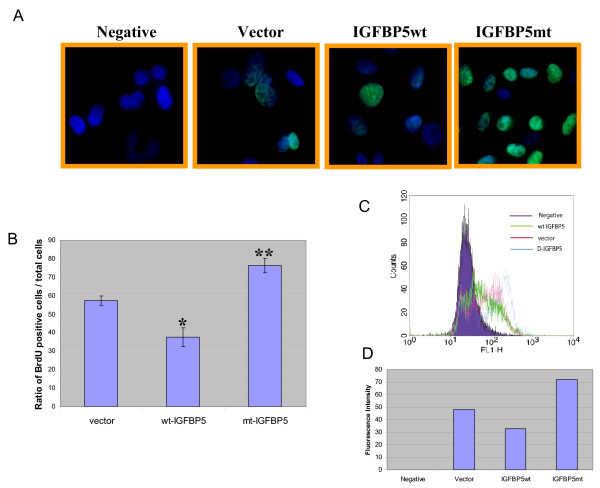
**Changes of IGFBP5 cellular distribution promotes breast cancer cell proliferation**. The cell growth rates for the vector and IGFBP5 transfectants were measured by BrdU incorporation using the BrdU flow kits per the manufacturer's instruction. **A**. The cells were incubated in 10% FBS medium containing 10 μM BrdU for 2 h followed by immunofluorescence staining with FITC conjugated BrdU antibody. The nuclei were counterstained with DAPI and images were taken at a magnification of 63×. Non-BrdU-treated parental cells were served as a negative control. **B**. Quantification data from 3 fields of each group. The total cells and the BrdU-positive cells were counted from 3 fields of each group, and the data were graphed as a ratio of BrdU positive cells vs. the total cells. The error bars represents standard deviation from three counts of samples. *, p < 0.05 and **, p < 0.005.**C**. The geometric mean fluorescence intensity from the same cell population as in **A **was recorded by FACS. **D**. The fluorescence intensity shown in **B **is shown as a bar graph. The fluorescence intensity from the negative control is graphed as zero, and the fluorescence intensity from the rest of the samples is the value after the value of the fluorescence intensity is subtracted from the negative control.

To determine any proliferative effects of the mutant form of IGFBP5, we performed BrdU incorporation analyses by flow cytometry and fluorescence microscopy. The cells overexpressing wild-type or mutant IGFBP5 were cultured in the complete media for 24 h and then incubated in the fresh complete media with 10 μM of BrdU for 2 h. The vector alone transfectant was used as the control. The incorporated BrdU was visualized by immunofluorescence staining using an anti-BrdU antibody conjugated with fluorescein isothiocyanate. Cells with no anti-BrdU antibody treatment served as the negative controls for the immunostaining. More cells overexpressing the mutant form of IGFBP5 were positively stained compared with the vector and the wild-type IGFBP5-overexpressing cells (Figure 5A). To obtain quantification data from the assay, we randomly chose 3 fields to count the total cells and the BrdU positive cells, and the data were graphed as a ratio of BrdU positive cells to total cells. The result revealed that overexpression of wild-type IGFBP5 significantly inhibited BrdU incorporation compared with the vector transfectants (*, p < 0.05) (Figure 5B). In contrast, overexpression of the mutant IGFBP5 significantly promoted BrdU incorporation (**, p < 0.005) (Figure 5B). To confirm our findings, the geometric mean fluorescence intensity from the same cell populations after the immunofluorescence staining was recorded by FACS. The fluorescence intensity from the negative control was graphed as zero, and fluorescence intensity from the rest of samples was the value after the fluorescence intensity value was subtracted from the negative control. The results from FACS analysis showed that wild-type IGFBP5 inhibited cell growth compared with the vector transfectant and that the mutated form of IGFBP5 promoted cell growth compared with the vector transfectant (Figure 5C and 5D).

### Deletion of amino acids 214 to 218 of IGFBP5 enhanced breast cancer cell motility

**Figure 6:**
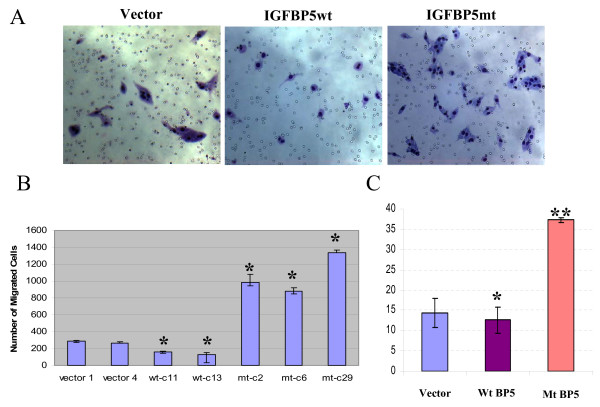
**Deletion of 5 amino acids from 214 to 218 of IGFBP5 protein promotes MDA-MB-435 breast cancer cell motility**. A. Representative light-microscopy images from the migration assay. B. Quantification data of *in vitro *migration assay from vector transfectants (vector 1 and vector 4), wild-type IGFBP5 overexpression clones (wt-c11 and wt-c13), and mutant IGFBP5 overexpression clones (mt-c2, mt-c6, and mt-c29). The error bars represent standard deviations from 2 separate experiments with triplicate samples for each (n = 6). *, p < 0.001. C. Quantification data of *in vitro *migration assay from transient transfection. The error bars represent standard deviations from triplicate samples. *, p < 0.05 and **, p < 0.001.

To determine whether mutation of NLS of IGFBP5 affects cell motility, we performed an *in vitro *migration assay using the stable cells overexpressing either wild-type or mutant IGFBP5. The vector-alone transfected cells were used as the control. As shown in Figure 6, the mutant IGFBP5-overexpressing cells had a significantly higher number of migrated cells than the vector-alone control cells (p < 0.01) (Figure 6A and 6B). In contrast, the wild-type IGFBP5-overexpressing cells had significantly lower numbers of migrated cells than the vector-alone control cells (p < 0.001) (Figure 6A and 6B). To confirm that deletion of amino acids 214 to 218 within the NLS of IGFBP5 promotes breast cancer cell motility, we transiently transfected the MDA-MB-435 breast cancer cells with IGFBP5wt/pcDNA3.1(+) and IGFBP5mt/pcDNA3.1(+) constructs and performed the *in vitro *migration assay 48-h post-transfection. The vector-alone transfected cells were used as the control. As shown in Figure 6C, we observed results similar to those from the *in vitro *migration experiments carried out using the stable cells. IGFBP5wt/pcDNA3.1(+)-transfected cells had a significantly lower number of migrated cells than the vector-alone control cells (* p < 0.05). In contrast, IGFBP5mt/pcDNA3.1(+)-transfected cells had a significantly higher number of migrated cells than the control cells (** p < 0.001) (Figure 6C).

## Discussion

There are contradictory findings and implications regarding whether IGFBP5 has enhancing and inhibitory effects on cells in breast cancer [6,13,20,23,24]. Clinical observations show that IGFBP5 is associated with the metastatic tumor phenotype in breast cancer, thus IGFBP5 is a poor prognostic factor and may serve as a target for therapeutic development [18,19,25,26]. The matter becomes complicated when attempting to understand the function of IGFBP5 in cell culture assays *in vitro*, studies have shown that IGFBP5 induces breast cancer cell apoptosis [13,20]. In this study, we provide evidence that the cellular localization of IGFBP5 is a key factor that affects its function in the cells. In primary cancer cells in tumor tissues, IGFBP5 is mainly located in the cytoplasm, whereas in cell culture, exogenously introduced IGFBP5 is mainly localized in the nucleus using its nuclear localization signal. When we deleted this signal from the IGFBP5, the function of IGFBP5 is switched from a growth inhibitory one to a growth and migration stimulating one, which is consistent with clinical observation that cytoplasmic IGFBP5 as a poor prognostic factor.

Recently, Jurgeit and colleagues also analyzed cytoplasmic trafficking and cellular localization of IGFBP5 in T47D breast cancer cell line and in paraffin sections of involuting mammary glands [27]. They examined the secretion and re-uptake of IGFBP5 of different mutant forms of the protein that either deleted signal peptide or NLS. They reported that that cellular localization of IGFBP5 was affected by secretion. They observed that the endogenous IGFBP5 was secreted into extracellular compartment via intracellular vesicles, taken up by cells, and then transported to vesicular compartments outside the nucleus. They suggested that wild-type IGFBP5 could not be localized in the nucleus under *in vivo *conditions and nuclear location of IGFBP5 was dependent on NLS. Jurgeit's findings in principle agree with our findings that IGFBP5 change cellular localization under different conditions although these conditions are yet to be clearly identified. Our studies have some different results, which resulted from different mutations in the NLS. We deleted the 214–218 (RGRKR) amino acids in NLS of IGFBP5, whereas Jurgeit and colleagues changed amino acids from RGRKR to LNGQL. It is not clear why the two different types of alteration generated some different phenotypes, but it is clear the NLS localization is critical for IGFBP5 function and our study showed that prevention of nuclear localization of IGFBP5 altered its function in the cells in a fashion that is consistent with *in vivo *clinical observation of IGFBP5.

However, deletion of the nuclear localization signal (NLS) from IGFBP5 does not occur *in vivo*. Thus, the studies using the deletion mutants represent a somewhat artificial condition although it is suggestive of potential mechanisms. Thus, a yet-to-be identified mechanism causing cytoplasmic localization of IGFBP5 operates in breast cancer tissues. It is conceivable that IGFBP5 in breast cancer tissues binds to another protein that modifies its conformation and deletion of the NLS has a similar effect on the conformation, which may prove to be important for IGFBP5 localization and function.

There is a precedent in p53 tumor suppressor protein for such a mechanism to operate in breast cancer. The tumor suppressor protein p53 is normally localized in the nucleus, where it functions as a transcriptional factor for genes involved in cell growth inhibition and apoptosis induction. Inactivation of the p53 tumor suppressor gene as the result of a mutation is one of the most recognized changes in cancer, with about 50% of cancers having such a mutation. Although mutated p53 is present in the nucleus, the mutations render it unable to bind to DNA and activate gene transcription [28]. In the other 50% of tumors, p53 is present and has a wild-type sequence. However, in these cases, the p53 pathway is inactivated through other means. One means is through the subcellular localization of the protein. p53 was first found in the cytoplasm in many inflammatory breast cancers [29] and then later in other tumors such as neuroblastomas [30]. The cytoplasmic localization of this wild-type p53 has now been associated with resistance to chemotherapy and radiation therapy and with a poor prognosis [31,32]. It was proposed that various proteins, including ribosomal proteins, vimentin, and tubulin, were involved in the abnormal localization of p53 in the cytoplasm [33-35]. Recently, an anchoring protein called Parc, or p53-associated parkin-like cytoplasmic protein [36], was found to bind p53 at the nuclear localization domains of p53 and attenuates the function of p53. The inactivation of endogenous Parc by siRNA was observed to restore the nuclear localization of p53 and sensitized the cells to DNA damage-induced cell death [37].

In our next investigation, we will determine whether Parc is the key regulator that renders IGFBP5 cytoplasmic in breast cancer cells in tumors and contributes to the growth and migration stimulatory effect. If Parc turns out not to be the regulator, identification of a new IGFBP5 regulator in the cells will certainly opens a new window into the pathogenesis of breast cancer biology. Interestingly, a recent report [38] suggests that IGFBP-5 regulates vascular cellular senescence via a p53-dependent pathway, thus the link between p53 and IGFBP5 may turn out to be closer than currently understood.

## Conclusion

The present study showed that deletion of 5 amino acids (214 to 218, RGRKR) within the NLS of IGFBP5 resulted in change in subcellular location of IGFBP5 and increased proliferation and motility of MDA-MB-435 breast cancer cells. This study provides supporting evidence that cellular localization of IGFBP5 is important for its functions in breast cancer.

## Abbreviations

IGFBP5: insulin-like growth factor binding protein-5; IGF: insulin-like growth factor; NLS: nuclear localization sequence; HBM: heparin-binding motif; BrdU: bromodeoxyuridine; FACS: fluorescence-activity cell sorter; FITC: fluorescein isothiocyanate.

## Competing interests

The authors declare that they have no competing interests.

## Authors' contributions

Both MA and LH performed the experimental studies and the statistical analysis, and they participated equally in drafting the manuscript. AS and XH contributed to the design of the study and interpretation of results; WZ conceived the study, contributed to the design of the study and interpretation of results, and worked on the manuscript. All authors read and approved the final manuscript.

## Pre-publication history

The pre-publication history for this paper can be accessed here:

http://www.biomedcentral.com/1471-2407/9/103/prepub
